# SARS-CoV-2 Serial Interval Variation, Montana, USA, March 1–July 31, 2020

**DOI:** 10.3201/eid2705.204663

**Published:** 2021-05

**Authors:** Isaiah G. Reed, Ethan S. Walker, Erin L. Landguth

**Affiliations:** Montana Department of Public Health and Human Services, Helena, Montana, USA (I.G. Reed);; University of Montana, Missoula, Montana, USA (E.S. Walker, E.L. Landguth)

**Keywords:** SARS-CoV-2, COVID-19, coronavirus, 2019 novel coronavirus disease, severe acute respiratory syndrome coronavirus 2, zoonoses, coronavirus disease, viruses, epidemics, infectious disease transmission, epidemiology, mathematical model, communicable disease control, rural population, coronavirus, basic reproduction number, Montana

## Abstract

We report mean severe acute respiratory syndrome coronavirus 2 serial intervals for Montana, USA, from 583 transmission pairs; infectors’ symptom onset dates occurred during March 1–July 31, 2020. Our estimate was 5.68 (95% CI 5.27–6.08) days, SD 4.77 (95% CI 4.33–5.19) days. Subperiod estimates varied temporally by nonpharmaceutical intervention type and fluctuating incidence.

In support of efforts in response to the emergence of severe acute respiratory syndrome coronavirus 2 (SARS-CoV-2), the pathogen causing novel coronavirus disease (COVID-19), the scientific community has attempted to predict its transmission trends, often through disease modeling. However, disease-specific parameter estimates for SARS-CoV-2 vary greatly. These parameters include the serial interval (SI), or the duration between onset of symptoms in connected primary and secondary cases, which is crucial in estimating epidemic reproduction numbers (R_0_) and assessing the effects of nonpharmaceutical interventions (NPIs) on transmission (*1*). Recent studies report SARS-CoV-2 SIs ranging from 2.97 to 7.5 days, with estimates representing primarily densely populated and urban settings (Table 1; Figure 1). The rural United States was relatively untouched in early epidemic waves, but major outbreaks followed in subsequent waves, so it is unknown whether rural- and urban-based transmission differ. Our objective was to report and compare SARS-CoV-2 SI values for Montana, USA, a primarily rural population, with other global and urban estimates. The study was defined as a public health surveillance activity by the University of Montana Institutional Review Board.

**Table 1:** Published mean serial interval estimates for severe acute respiratory syndrome coronavirus 2*

Publication†	Study location, dates (all in 2020 except as indicated)	No. cases (pairs)	SI mean (95% CI)	SI SD (95% CI)	SI estimate method
This study	Montana, USA, Mar 1–Jul 31	4,793 (583)	5.68 (5.27–6.08)	4.77 (4.33–5.19)	Forward
Prete et al., 2020 (*13*)‡	Brazil, Feb 25–Mar 19	NA (65)	2.97	3.29	Other
Talmoudi et al., 2020 (*14*)‡	Tunisia, Feb 29–May 5	NA (491)	5.30 (4.66–5.95)	0.26 (0.23–0.30)	Other
Lavezzo et al., 2020 (*15*)	Vo’, Italy, Feb 21–Mar 7	81 (41)	7.2 (5.9–9.6)	NA	Other
Aghaali et al., 2020 (*16*)	Qom, Iran, Feb 20–Mar 8	88 (37)	4.55	3.30	Forward
You et al., 2020 (*17*)‡	China (OHP), as of Mar 31	14,828 (198)	4.60	5.55	Intrinsic
Ali et al., 2020 (*1*)‡	China (OHP), Jan 9–Feb 13	9,120 (677)	5.1 (4.7–5.5)	5.3 (5.0–5.6)	Forward
Zhang et al., 2020 (*18*)	China (OHP), Jan 19–Feb 17	8,579 (35)	5.1 (1.3–11.6)	NA	Forward
Du et al., 2020 (*10*)‡	China (OHP), Jan 21–Feb 8	752 (468)	3.96 (3.53–4.39)	4.75 (4.46–5.07)	Backward
Liao et al., 2020 (*19*)	China (CTGCH), Jan 7–Mar 20	46 (12)	6.50 (2.45–17.38)	NA	Forward
Zhao et al., 2020 (*20*)	Hong Kong, Jan 16–Feb 15	56 (21)	4.9 (3.6–6.2)	4.4 (2.9–8.3)	Other
Chan et al., 2020 (*21*)	Hong Kong, Jan 23–Apr 6	915 (47)	6.5 (0–18)	4.7	Unknown
Bi et al., 2020 (22)	Shenzhen, China, Jan 14–Feb 9	391 (48)	6.3 (5.2–7.6)	4.2 (3.1–5.3)	Other
Wang et al., 2020 (23)	Shenzhen, China, Jan 19–Feb 22	417 (27)	5.9 (3.9–9.6)	4.8 (3.1–10.1)	Other
Ganyani et al., 2020 (24)‡	Tianjin, China, Jan 14–Feb 27	135 (NA)	3.95 (–4.47 to 12.51)	4.24 (4.03–4.95)	Other
Tindale et al., 2020 (25)	Tianjin, China, Jan 21–Feb 22	135 (72)	4.31 (2.91–5.72)	0.716	Forward
Li et al., 2020 (26)	Wuhan, China, as of Jan 22	425 (6)	7.5 (5.3–19.0)	3.4	Other
Ganyani et al., 2020 (24)‡	Singapore, Jan 21–Feb 26	91 (NA)	5.21 (–3.35 to 13.94)	4.32 (4.06–5.58)	Other
Tindale et al., 2020 (25)	Singapore, Jan 23–Feb 26	93 (56)	4.17 (2.44–5.89)	0.882	Forward
Ki et al., 2020 (27)	South Korea, Jan 10–Feb 10	28 (12)	6.6 (3–15)	NA	Unknown
Mettler et al., 2020 (*12*)‡	South Korea, Jan 20–Jun 30	5,201 (102)	3.43 (2.62–4.24)	NA	Forward
Chun et al., 2020 (28)‡	South Korea, Jan 23–Mar 31	9,887 (69)	3.18 (2.22–4.24)	0.75 (0.47–1.03)	Forward
Son et al., 2020 (29)	Busan, South Korea, Feb 21–Mar 24	108 (28)	5.54 (4.08–7.01)	3.90 (2.47–5.32)	Other
Nishiura et al., 2020 (30)	Meta-analysis, 2019 Dec 21–2020 Feb 12	NA (28)	4.7 (3.7–6.0)	2.9 (1.9–4.9)	Other
He et al., 2020 (*11*)‡	Meta-analysis, Jan 21–Feb 12	NA (77)	5.8 (4.8–6.8)	NA	Other

*All articles published during 2020 except this study. CTGCH, Chongqing Three Gorges Central Hospital; NA, not available; OHP, outside Hubei Province; SARS-CoV-2, severe acute respiratory syndrome coronavirus 2; SI, serial interval. †See References and Appendix for full publication information. ‡Study included negative-valued serial interval pairs in the estimate.

**Figure 1:**
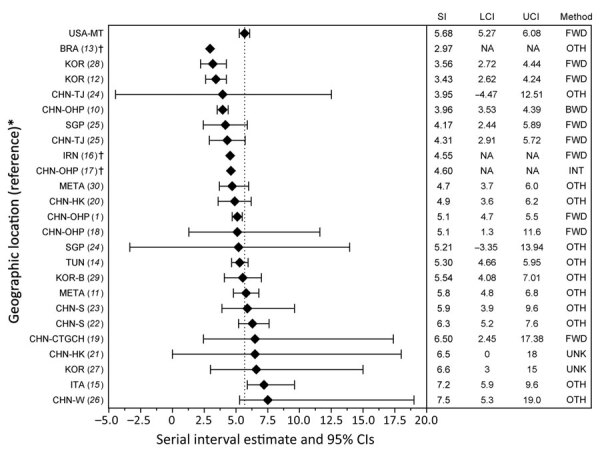
Published mean serial interval estimates for severe acute respiratory syndrome coronavirus 2. *See References and Appendix for full study information. †These studies did not report CIs. Only point estimates are given. BRA, Brazil; BWD, backward; CHN-CTGCH, China–Chongqing Three Gorges Central Hospital; CHN-HK, China–Hong Kong; CHN-OHP, China–outside Hubei Province; CHN-S, China–Shenzhen; CHN-TJ, China–Tianjin; CHN-W, China–Wuhan; FWD, forward; INT, intrinsic; IRN, Iran; ITA, Italy; KOR, South Korea; KOR-B, South Korea–Busan; LCI, lower confidence interval; META, meta-analysis; NA, data not available; OTH, other; SGP, Singapore; TUN, Tunisia; UCI, upper confidence interval; UNK, unknown; USA-MT, United States–Montana.

## The Study

We acquired COVID-19 data, reported by local health jurisdictions, from the Montana Department of Public Health and Human Services; we obtained 45,102 case records as of November 15, 2020. We examined a subset of cases with symptom onset dates during March 1–July 31, 2020 (n = 4,793), as well as secondary cases resulting from primary infections during that period, regardless of onset date. We selected this period because all reported cases were PCR positive, all NPI stages were represented (pre–shelter-in-place [pre-SIP], shelter-in-place [SIP], and reopening phase 1 and phase 2), and the proportion of identifiable transmission chains among cases was relatively high (March–June 39%–44%; July 11%) compared with later periods (August–November 0%–2%).

We assessed the records to identify all epidemiologic links. We defined links as cases having contact with another reported case, when viral infection through accepted modes of transmission was plausible. Linked records (n = 1,005) were organized into pairs and designated as primary or secondary cases. When appropriate, cases were listed as primaries for multiple secondary cases; however, cases were limited to 1 secondary designation. For some secondary cases, 1 specific primary was not clearly defined. To estimate a serial range in these situations, we assigned upper and lower bounds using the shortest and longest SIs from all possible primaries. We excluded records when we could not determine an epidemiologic link or transmission direction. We identified 583 pairs, with 466 primary and 583 secondary cases.

We gave temporal markers to pairs on the basis of the primary case’s symptom onset date, consistent with forward-looking SIs (*2*), and grouped them by the corresponding statewide NPI: pre-SIP, March 1–27; SIP, March 28–April 25; phase 1, April 26–May 31; phase 2 (June), June 1–30; and phase 2 (July), July 1–31. We divided phase 2 into 2 subperiods to account for changing incidence trends.

We analyzed data using R version 3.6.2 and the EpiEstim package (*3*,*4*). Complying with EpiEstim functional requirements, we assigned pairs with a zero-valued SI an upper bound of 1 day, with lower bounds unchanged (n = 52 pairs). No negative-valued SIs were identified. We excluded pairs with a SI >2 incubation periods (>28 days). We determined that a gamma distribution was most appropriate using the R0 package *est.GT* function (*5*). Next, we used EpiEstim *estimate_R*, with case-pair and daily incidence data, to perform a Bayesian estimation of the SI gamma distribution using Markov chain Monte Carlo specified for the joint posterior sample of possible SI values (*6*,*7*).

**Figure 2:**
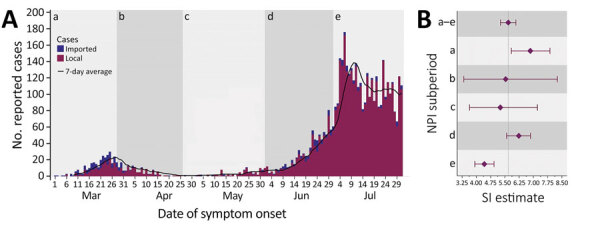
Reported COVID-19 cases and SARS-CoV-2 SI estimates by NPI subperiod, Montana, USA, March 1–July 31, 2020. A) COVID-19 cases, by date of symptom onset. Total cases, 4,793; total pairs, 583. For subperiod pair totals, see the Forward section of Table 2. B) SI estimates and 95% CIs (error bars). Overall mean SI was 5.68 (95% CI 5.27–6.08) days, overall SD 4.77 (95% CI 4.33–5.19) days. For subperiod SI and SD estimates, see the Forward section of Table 2. SI estimates are forward-looking and are based on the symptom onset date of the primary case in the infector–infectee pair. NPI subperiods: a) Pre-SIP, March 1–27, no NPIs in place; no. cases, 285. b) SIP, March 28–April 25, statewide stay-at-home order instituted and all nonessential businesses closed; no. cases, 168. c) Phase 1, April 26–May 31, statewide stay-at-home order lifted and limited business types allowed to open with reduced capacity; no. cases, 99. d) Phase 2 (June), June 1–30, all business types allowed to open under less restrictive capacity regulations; no. cases, 824. e) Phase 2 (July), July 1–31, all business types allowed to open under less restrictive capacity regulations; no. cases 3,417. Black line is the average number of cases for the preceding 7 days. Imported case: COVID-19 case linked to out-of-state OR out-of-county transmission; local case: nonimported COVID-19 case linked to in-state AND in-county transmission. COVID-19, coronavirus disease 2019; SARS-CoV-2, severe acute respiratory syndrome coronavirus 2; SI, serial interval; SIP, shelter-in-place.

Montana’s overall mean SI estimate was 5.68 (95% CI 5.27–6.08) days (SD 4.77 [95% CI 4.33–5.19] days) (Figure 2). Pre-SIP provided the longest subperiod estimate, 6.84 (95% CI 5.84–7.87) days. The SI shortened during SIP, to 5.54 (95% CI 3.34–8.26) days, and again during phase 1, to 5.26 (95% CI 3.64–7.21) days. However, the SI lengthened during phase 2 (June) to 6.23 (95% CI 5.59–6.85) days, almost reaching pre-SIP levels. Phase 2 (July) demonstrated a sharp reduction to the shortest SI observed, 4.42 (95% CI 3.92–4.93) days. Sensitivity analyses of NPI impact delays resulted in altered subperiod estimates, especially for phase 1 relative to other subperiods (Table 2). Additional sensitivity analyses, comparing forward- and backward-looking SIs, produced vastly dissimilar point estimates and trends.

**Table 2:** Sensitivity analyses: forward and backward severe acute respiratory syndrome coronavirus 2 serial interval estimates by nonpharmaceutical intervention subperiod and length of intervention effects delay*

SI estimate method	NPI subperiod	Measure	Sensitivity analysis scenarios†		
			No delay	1-week delay	2-week delay
Forward: onset of primary case	Pre–shelter-in-place, Mar 1–27	No. pairs	95	105	113
		Mean SI (95%CI)	6.84 (5.84–7.87)	6.83 (5.67–8.07)	6.66 (5.61–7.80)
		SD (95% CI)	5.56 (4.45–6.80)	5.78 (4.48–7.24)	5.61 (4.50–6.84)
	Shelter-in-place, Mar 28–Apr 25	No. pairs	20	10	3
		Mean SI (95% CI)	5.54 (3.34–8.26)	4.08 (2.61–5.85)	2.46 (1.24–4.10)
		SD (95% CI)	5.30 (2.69–8.76)	2.83 (1.47–4.66)	1.52 (0.38–3.38)
	Reopening, phase 1, Apr 26–May 31	No. pairs	25	64	114
		Mean SI (95% CI)	5.26 (3.64–7.21)	7.45 (6.02–9.02)	7.10 (6.08–8.16)
		SD (95% CI)	4.74 (2.86–7.09)	6.24 (4.70–8.03)	5.82 (4.77–6.99)
	Reopening, phase 2, Jun 1–30	No. pairs	248	296	289
		Mean SI (95% CI)	6.23 (5.59–6.85)	5.39 (4.88–5.94)	5.08 (4.56–5.59)
		SD (95% CI)	5.32 (4.61–6.05)	4.59 (4.01–5.21)	4.32 (3.75–4.94)
	Reopening, phase 2, Jul 1–31	No. pairs	195	117	76
		Mean SI (95% CI)	4.42 (3.92–4.93)	4.20 (3.65–4.78)	3.98 (3.36–4.67)
		SD (95% CI)	3.51 (2.97–4.06)	3.20 (2.65–3.80)	2.90 (2.29–3.60)
Backward: onset of secondary case	Pre–shelter-in-place, Mar 1–27	No. pairs	61	89	105
		Mean SI (95% CI)	4.82 (3.88–5.84)	5.83 (4.86–6.82)	6.48 (5.55–7.51)
		SD (95% CI)	3.84 (2.88–4.93)	4.91 (3.86–6.08)	5.50 (4.44–6.63)
	Shelter-in-place, Mar 28–Apr 25	No. pairs	54	26	11
		Mean SI (95% CI)	8.57 (6.77–10.58)	9.03 (6.73–11.66)	7.58 (4.29–11.83)
		SD (95% CI)	6.95 (5.10–8.99)	6.52 (4.28–9.22)	6.21 (2.91–10.73)
	Reopening, phase 1, Apr 26–May 31	No. pairs	19	30	62
		Mean SI (95% CI)	3.79 (2.46–5.37)	4.95 (3.53–6.60)	4.57 (3.64–5.60)
		SD (95% CI)	3.10 (1.70–4.90)	4.41 (2.78–6.43)	3.73 (2.72–4.90)
	Reopening, phase 2, Jun 1–30	No. pairs	202	280	310
		Mean SI (95% CI)	5.38 (4.72–6.08)	5.14 (4.64–5.67)	5.22 (4.73–5.77)
		SD (95% CI)	4.59 (3.86–5.41)	4.31 (3.77–4.90)	4.38 (3.85–4.97)
	Reopening, phase 2, Jul 1–31	No. pairs	233	161	106
		Mean SI (95% CI)	5.43 (4.85–6.05)	5.82 (5.12–6.56)	6.45 (5.37–7.57)
		SD (95% CI)	4.52 (3.90–5.17)	4.88 (4.14–5.70)	5.41 (4.35–6.64)

*NPI, nonpharmaceutical intervention; SI, serial interval. †Serial interval estimation methods and delay scenarios contain dissimilar pair totals because of their temporal differences (forward pairs, n) no delay: 583; 1-week delay: 592; 2-week delay: 595; (backward pairs, n) no delay: 569; 1-week delay: 586; 2-week delay: 594.

## Conclusions

Analysis of SARS-CoV-2 transmission in Montana during March 1–July 31, 2020, identified a mean SI of 5.68 (95% CI 5.27–6.08) days, falling within the bounds of 16 of 24 published estimates from more urbanized settings across the globe (Table 1; Figure 1). However, an aggregate estimate derived from data spanning multiple outbreak stages may not accurately describe Montana-based transmission because changing contact patterns and environmental influences may cause variation (*1*,*2*). Temporal analyses suggest that NPIs influenced transmission patterns, as demonstrated by Montana’s epidemic curve and fluctuating SI values (Figure 2). Ali found that SIs shorten as stricter NPIs are applied (*1*,*8*), which our subperiod estimates mostly support. However, phase 2 (July) contradicts the premise, with the shortest subperiod SI and a less restrictive NPI (Table 2). Furthermore, when accounting for NPI impact delays, the alignment falters during phase 2. This difference may occur because Ali did not assess additional epidemic waves, which complicates direct NPI comparisons (*1*). Park agreed with Ali, while also offering a mathematical proof for the relationship between epidemic growth rates, calculated from incidence data, and forward-looking SIs (*2*,*9*). Park showed that as growth rates increase, forward SIs lengthen, and that when incidence decreases (either over time or because of external factors) forward SIs shorten (*2*). This better describes Montana’s incidence and our subperiod estimates, with NPIs providing context (Figure 2). Increased incidence and longer SIs during pre-SIP and phase 2 (June) stem from nonexistent and relaxed NPIs, whereas decreased incidence and shorter SIs during SIP and phase 2 (July) likely result from stricter NPIs and increased compliance with public health recommendations (e.g., mask wearing and social distancing). Additional data describing social compliance would benefit this interpretation.

The first limitation of this study is that the proportion of cases with identifiable transmission chains was lower during July than in previous periods. Despite this limitation, we felt it was necessary to report an SI for a period experiencing sizable incidence fluctuations. In addition, whereas others have reported negative-valued SIs among 1.2%–14.46% of infector–infectee pairs (*10**–**14*), we failed to identify any within our data. This difference could be caused by multiple factors, including incorrectly reported symptom onset dates, misidentified transmission direction between pairs, or both. However, the absence of negative SIs was not unique to our study; 14 of 24 published SI estimates did not include negative-valued pairs (Table 1).

Furthermore, to include pairs with a zero-valued SI, our study required changing their upper range. A sensitivity analysis of the adjustment showed minimal impact to the resulting estimate, whereas another sensitivity analysis, examining zero-valued pairs’ exclusion, returned a substantially elevated estimate. These analyses indicate that nontraditional SIs play key roles in generation time, SI, and R_0_ studies, especially for SARS-CoV-2, and that inclusive methods should be used when possible.

Our study offers evidence that rural-based SARS-CoV-2 SI estimates are consistent with those describing transmission occurring in urban settings. Furthermore, temporal variations in incidence, which can be caused by NPIs, must be considered when assessing SI distributions and other transmission measures. More period-based analyses of varying NPIs and their effects on transmission dynamics would help corroborate these findings.

Appendix Additional references for study of SARS-CoV-2 serial infection variation, Montana.
