# Supplementary material for: SARS-CoV-2 Serial Interval Variation, Montana, USA, March 1–July 31, 2020
Source: Emerg Infect Dis. 2021 May;27(5):1486–91. doi: 10.3201/eid2705.204663 (PMC8084495; doi:10.3201/eid2705.204663)
Supplement: Appendix — Additional references for study of SARS-CoV-2 serial infection variation, Montana. [file 20-4663-Techapp-s1.pdf]

Article DOI: https://doi.org/10.3201/eid2705.204663

Severe Acute Respiratory Syndrome Coronavirus 2 Serial Interval Variation, Montana, USA, March 1–July 31, 2020

Appendix

Additional References, Table 1 and Figure 1

15. Lavezzo E, Franchin E, Ciavarella C, Cuomo-Dannenburg G, Barzon L, Del Vecchio C, et al.; Imperial College COVID-19 Response Team; Imperial College COVID-19 Response Team. Suppression of a SARS-CoV-2 outbreak in the Italian municipality of Vo'. Nature. 2020;584:425–9. PubMed https://doi.org/10.1038/s41586-020-2488-1

16. Aghaali M, Kolifarhood G, Nikbakht R, Saadati HM, Hashemi Nazari SS. Estimation of the serial interval and basic reproduction number of COVID-19 in Qom, Iran, and three other countries: A data-driven analysis in the early phase of the outbreak. Transbound Emerg Dis. 2020;67:2860–8. PubMed https://doi.org/10.1111/tbed.13656

17. You C, Deng Y, Hu W, Sun J, Lin Q, Zhou F, et al. Estimation of the time-varying reproduction number of COVID-19 outbreak in China. Int J Hyg Environ Health. 2020;228:113555. PubMed https://doi.org/10.1016/j.ijheh.2020.113555

18. Zhang J, Litvinova M, Wang W, Wang Y, Deng X, Chen X, et al. Evolving epidemiology and transmission dynamics of coronavirus disease 2019 outside Hubei province, China: a descriptive and modelling study. Lancet Infect Dis. 2020;20:793–802. PubMed https://doi.org/10.1016/S1473-3099(20)30230-9

19. Liao J, Fan S, Chen J, Wu J, Xu S, Guo Y, et al. Epidemiological and clinical characteristics of COVID-19 in adolescents and young adults. Innovation (N Y). 2020;1:100001. PubMed https://doi.org/10.1016/j.xinn.2020.04.001

20. Zhao S, Gao D, Zhuang Z, Chong MKC, Cai Y, Ran J, et al. Estimating the serial interval of the novel coronavirus disease (COVID-19): a statistical analysis using the public data in Hong Kong from January 16 to February 15, 2020. Front Phys. 2020;8:347. https://doi.org/10.3389/fphy.2020.00347

21. Chan YWD, Flasche S, Lam TLT, Leung MHJ, Wong ML, Lam HY, et al. Transmission dynamics, serial interval and epidemiology of COVID-19 diseases in Hong Kong under different control measures [version 2; peer review: 2 approved with reservations]. Wellcome Open Res. 2020;5:91. https://doi.org/10.12688/wellcomeopenres.15896.1

22. Bi Q, Wu Y, Mei S, Ye C, Zou X, Zhang Z, et al. Epidemiology and transmission of COVID-19 in 391 cases and 1286 of their close contacts in Shenzhen, China: a retrospective cohort study. Lancet Infect Dis. 2020;20:911–9. PubMed https://doi.org/10.1016/S1473-3099(20)30287-5

23. Wang K, Zhao S, Liao Y, Zhao T, Wang X, Zhang X, et al. Estimating the serial interval of the novel coronavirus disease (COVID-19) based on the public surveillance data in Shenzhen, China, from 19 January to 22 February 2020. Transbound Emerg Dis. 2020;67:2818–22. PubMed https://doi.org/10.1111/tbed.13647

24. Ganyani T, Kremer C, Chen D, Torneri A, Faes C, Wallinga J, et al. Estimating the generation interval for coronavirus disease (COVID-19) based on symptom onset data, March 2020. Euro Surveill. 2020;25:2000257. PubMed https://doi.org/10.2807/1560-7917.ES.2020.25.17.2000257

25. Tindale LC, Stockdale JE, Coombe M, Garlock ES, Lau WYV, Saraswat M, et al. Evidence for transmission of COVID-19 prior to symptom onset. eLife. 2020;9:e57149. PubMed https://doi.org/10.7554/eLife.57149

26. Li Q, Guan X, Wu P, Wang X, Zhou L, Tong Y, et al. Early Transmission Dynamics in Wuhan, China, of Novel Coronavirus-Infected Pneumonia. N Engl J Med. 2020;382:1199–207. PubMed https://doi.org/10.1056/NEJMoa2001316

27. Ki M; Task Force for 2019-nCoV. Epidemiologic characteristics of early cases with 2019 novel coronavirus (2019-nCoV) disease in Korea. Epidemiol Health. 2020;42:e2020007. PubMed https://doi.org/10.4178/epih.e2020007

28. Chun JY, Baek G, Kim Y. Transmission onset distribution of COVID-19. Int J Infect Dis. 2020;99:403–7. PubMed https://doi.org/10.1016/j.ijid.2020.07.075

29. Son H, Lee H, Lee M, Eun Y, Park K, Kim S, et al. Epidemiological characteristics of and containment measures for COVID-19 in Busan, Korea. Epidemiol Health. 2020;42:e2020035. PubMed https://doi.org/10.4178/epih.e2020035

30. Nishiura H, Linton NM, Akhmetzhanov AR. Serial interval of novel coronavirus (COVID-19) infections. Int J Infect Dis. 2020;93:284–6. PubMed https://doi.org/10.1016/j.ijid.2020.02.060
